# Draft Genome Sequence of *Dysgonomonas macrotermitis* Strain JCM 19375^T^, Isolated from the Gut of a Termite

**DOI:** 10.1128/genomeA.00963-15

**Published:** 2015-08-27

**Authors:** Xinxin Sun, Yingjie Yang, Ning Zhang, Yulong Shen, Jinfeng Ni

**Affiliations:** State Key Laboratory of Microbial Technology, Shandong University, Jinan, Shandong, China

## Abstract

Here, we report the draft genome sequence of *Dysgonomonas macrotermitis* strain JCM 19375^T^, which was isolated from the hindgut of a fungus-growing termite, *Macrotermes barneyi*. The genome information reveals the role of this strain in lignocellulose degradation and adaptation to the gut environment.

## GENOME ANNOUNCEMENT

The termite contains various microorganisms that symbiotically exist in the hindgut. Metagenomic analysis has revealed a large diverse set of glycoside hydrolyase (GH) genes presenting in the hindgut of a higher termite, which may play an important role in lignocellulose degradation (1). The genus *Dysgonomonas* comprises seven species with validly published names: *D. gadei*, *D. capnocytophagoides*, *D. mossii*, *D. hofstadii*, *D. oryzarvi*, *D. termitidis*, and *D. macrotermitis* (2–8). The first four species are all from humans, and the latter three are from microbial fuel cell and termite guts. *D. macrotermitis*, a novel species of the genus *Dysgonomonas*, is the second most dominant bacterium in the hindgut of a fungus-growing termite, *Macrotermes barneyi* (8). To investigate the symbiotic roles of the strain, its genome sequence was analyzed.

The genome of the strain was sequenced using Illumina MiSeq. A total of 2,809,508 reads were assembled into 78 contigs, with an *N*_50_ length of 149,547 bp, and the largest contig length was 790,807 bp. This assembly resulted in a draft genome sequence of 4,655,756 bp and a G+C content of 38.54%. Together, they contain 3,870 coding sequences (CDSs), 44 tRNA genes, and 3 complete rRNA operons.

We assessed the potential role of *D. macrotermitis* in lignocellulose digestion by screening the CDSs against the CAZy database (9). Preliminary analyses revealed the presence of genes for cellulolytic and hemicellulolytic enzymes in the genome sequence of *D. macrotermitis*. There were various genes encoding glucosidase of glycoside hydrolase 2 (GH2), GH3, and GH97; genes encoding glucanase of GH5, -16, and -26; genes encoding xylanases of the GH10, -11, and -43 families; genes encoding α- and β-xylosidases of GH43, -3, -39, and -31; and genes encoding α- and β-galactosidase of GH2, -27, -35, -36, -42, -43, -53, and -97. In addition, the genome had several genes coding α- and β- glucuronidase, arabinofuranosidase, arabinosidase, and amylase. These results suggest the potential role of the strain in decomposing lignocellulose and providing nutrition to the host termite in the hindguts of fungus-growing termites.

### Nucleotide sequence accession numbers.

This whole-genome shotgun project has been deposited in DDBJ under the accession numbers BBXL01000001 to BBXL01000078.
